# Single-Particle Plasmon Sensor to Monitor Proteolytic Activity in Real Time

**DOI:** 10.1021/acsaom.3c00226

**Published:** 2023-10-04

**Authors:** Rui Oliveira-Silva, Yuyang Wang, Sjoerd W. Nooteboom, Duarte M. F. Prazeres, Pedro M. R. Paulo, Peter Zijlstra

**Affiliations:** † MBx Molecular Biosensing, Department of Applied Physics and Institute for Complex Molecular Systems, Eindhoven University of Technology, P.O. Box 513, 5600 MB Eindhoven, The Netherlands; ‡ iBB − Institute for Biotechnology and Bioengineering, Instituto Superior Técnico, Universidade de Lisboa, 1049-001 Lisboa, Portugal; § Associate Laboratory i4HB—Institute for Health and Bioeconomy, Instituto Superior Técnico, Universidade de Lisboa, 1049-001 Lisboa, Portugal; ∥ CQE—Centro de Química Estrutural, Institute of Molecular Sciences, Instituto Superior Técnico, Universidade de Lisboa, Avenida Rovisco Pais 1, 1049-001 Lisboa, Portugal

**Keywords:** plasmonic nanoparticles, monitoring, proteolytic sensor, single-particle spectroscopy, thrombin, modeling, and kinetics

## Abstract

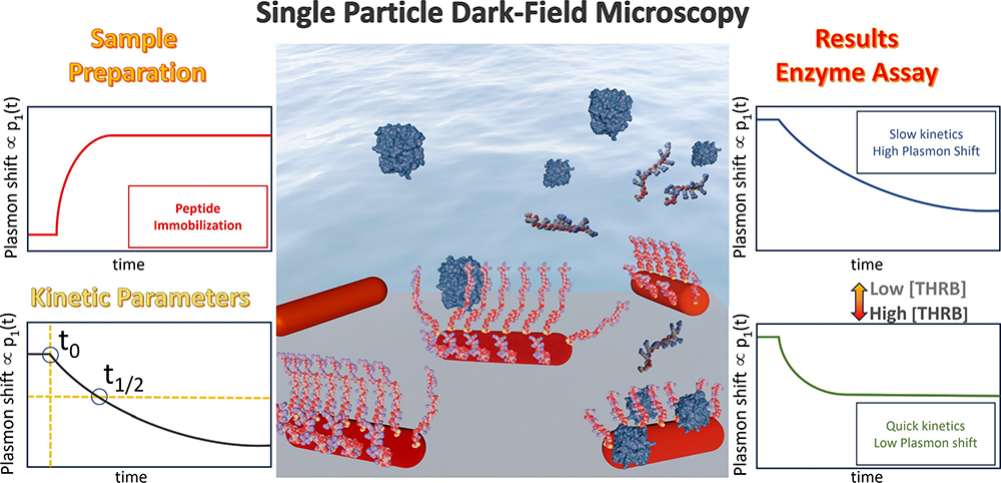

We have established a label-free plasmonic platform that monitors proteolytic activity in real time. The sensor consists of a random array of gold nanorods that are functionalized with a design peptide that is specifically cleaved by thrombin, resulting in a blueshift of the longitudinal plasmon. By monitoring the plasmon of many individual nanorods, we determined thrombin’s proteolytic activity in real time and inferred relevant kinetic parameters. Furthermore, a comparison to a kinetic model revealed that the plasmon shift is dictated by a competition between peptide cleavage and thrombin binding, which have opposing effects on the measured plasmon shift. The dynamic range of the sensor is greater than two orders of magnitude, and it is capable of detecting physiologically relevant levels of active thrombin down to 3 nM in buffered conditions. We expect these plasmon-mediated label-free sensors to open the window to a range of applications stretching from the diagnostic and characterization of bleeding disorders to fundamental proteolytic and pharmacological studies.

## Introduction

The plasmon resonance of metallic nanoparticles depends not only on the shape, size, and material of the particle but also on the local refractive index of its vicinity. This label-free detection has sparked the usage of plasmonic nanoparticles as biosensors as they can detect changes in the local refractive index due to the occurrence of biomolecular events, such as binding, that are transduced to shifts of the plasmon resonance.^1^ In addition, plasmonic nanoparticles are chemically inert and exhibit long-lasting photostability, which is highly beneficial in the monitoring of molecular interactions or processes. Plasmon sensors have been developed for a large range of analytes^2^ and with a large range of particle shapes and sizes.^3^ The majority of these sensors are based on suspensions of particles and essentially monitor the ensemble-averaged optical spectrum in a spectrometer. A major drawback of this approach is that the optical spectrum is inhomogeneously broadened because even the best nanoparticle synthesis methods yield size distributions of ∼5%.^4,5^

Recently, sensors based on single immobilized particles, rather than particle suspensions, have proven their advantage as they exhibit a higher sensitivity due to the absence of inhomogeneous broadening.^6^ The sensitivity of plasmon sensors is often quantified by a figure-of-merit (FoM),^7^ which is most often defined as the ratio between the refractive-index sensitivity (in eV/refractive-index unit) and resonance linewidth (in eV). Previous single-particle studies reported the FoM for isolated gold spheres and found values ranging from 0.5 to 1.^8^ Simulations of gold nanoshells reported a slightly higher FoM of 2.5.^9^ In general, elongated gold structures profit from an increased sensitivity as their tips get sharper, that combined with a narrow LSPR peak away from the interband transitions in gold results in improved sensing performance. Indeed, FoMs of ∼5 to 10 are typical for individual nanorods and bipyramids, which has led to the first demonstrations of plasmon biosensors with single-molecule sensitivity.^10−12^

To enable the tracking of the plasmon resonance of a single particle over time, the particles are usually immobilized onto a substrate at low density to enable long-term monitoring of the same set of particles. Furthermore, this implementation circumvents potential problems related to colloidal stability where plasmon shifts due to particle clustering cannot be distinguished from the desired signals. These features combined with shot-noise-limited dark-field microscopy.^12^ These platforms have been used to measure the concentration of biomolecules by quantifying the time-dependent redshift of the plasmon resonance.^13^

For enzymes such as proteases, however, gene expression and affinity-based approaches have demonstrated that it is not the concentration of enzyme but rather the enzymatic activity that renders crucial and accurate information about the system and its function.^14^ Colorimetric^15^ and mass-spectrometry techniques^16,17^ are commonly used to determine proteolytic activity. Nonetheless, these strategies often present limited sensitivity and specificity and do not enable real-time measurements. This has sparked developments in assays based on Förster Resonance Energy Transfer that demonstrated detection limits in the nanomolar range and enabled real-time measurements. However, the photostability of organic fluorophores is often compromised by environmental parameters such as pH, ionic strength, and the presence of redox species.

Here, we demonstrate a plasmonic platform that enables the real-time monitoring of proteolytic activity in a label-free manner. Compared to previous plasmonic sensors, the ability to monitor in real time is achieved by a combination of two approaches: the use of AuNRs provides 5-fold higher sensitivity compared to nanospheres,^18^ while sensing at the single-particle level eliminates inhomogeneous broadening further boosting the signal.^19^ The sensor optically probes the plasmon shift of hundreds of gold nanorods (AuNRs) simultaneously achieving a dynamic range of >2 orders of magnitude with a physiologically relevant detection limit of 3 nM. We present a kinetic model that describes the plasmon shift as a competition between peptide cleavage and thrombin binding, which have opposing effects on the measured plasmon shift. The use of a peptide-based substrate enables the generalization to other enzymes and the (multiplexed) detection of a panel of proteases or even hydrolases from other families.

We use thrombin as a model protease due to its central role in the coagulation cascade and its key role in clotting diseases.^20−23^ Also, its involvement in the homeostasis of the gut and central nervous system has been recently suggested,^24−27^ indicating its potential as a biomarker for other pathological conditions. Hence, biosensing assays to quantify thrombin’s activity are much needed in both fundamental biochemistry studies and in the future in clinical settings.

**Figure 1:**
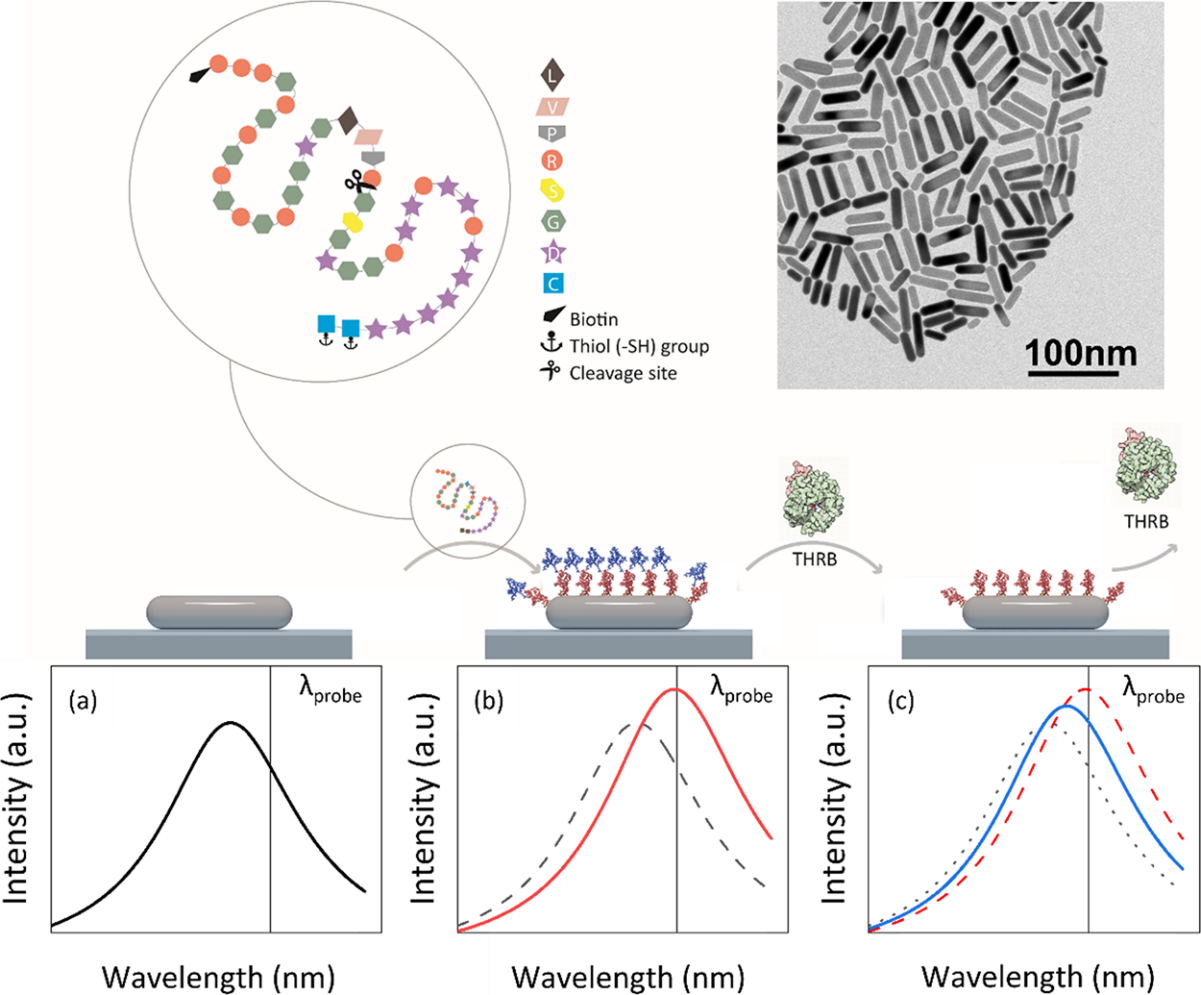
Schematic working principle of the plasmonic proteolytic sensor. The thrombin-specific peptide with the cleavage site is depicted, together with an electron microscopy image of a dried drop of the AuNRs on a carbon-coated grid. The bottom diagram illustrates the workflow of the sensor involving the peptide immobilization step and the sequential peptide cleavage by thrombin (THRB) along with the induced plasmon redshifts (b) and blueshifts (c). The plasmon shifts are probed in real time at the single-particle level.

The underlying principle of our single-particle proteolytic biosensor is schematically presented in Figure 1. AuNRs are immobilized on a coverslip by spin coating and then functionalized with a thiolated peptide that contains a thrombin-specific cleavage site in the middle of the sequence. The evanescent tail of the electromagnetic near-field associated with the plasmon decays on length scales of tens of nanometers.^28^ Refractive index changes in this near-field are transduced to shifts in the plasmon resonance.^12,29^ Upon coating the particle with the peptide (b), the plasmon resonance will therefore redshift, whereas subsequent introduction of active thrombin will result in a gradual blueshift of the plasmon due to cleavage of the conjugated peptide (c). These processes are probed in an optical microscope by monitoring the scattering signal of many individual particles in real time in a wide-field geometry.^12,30^

## Results and Discussion

**Figure 2:**
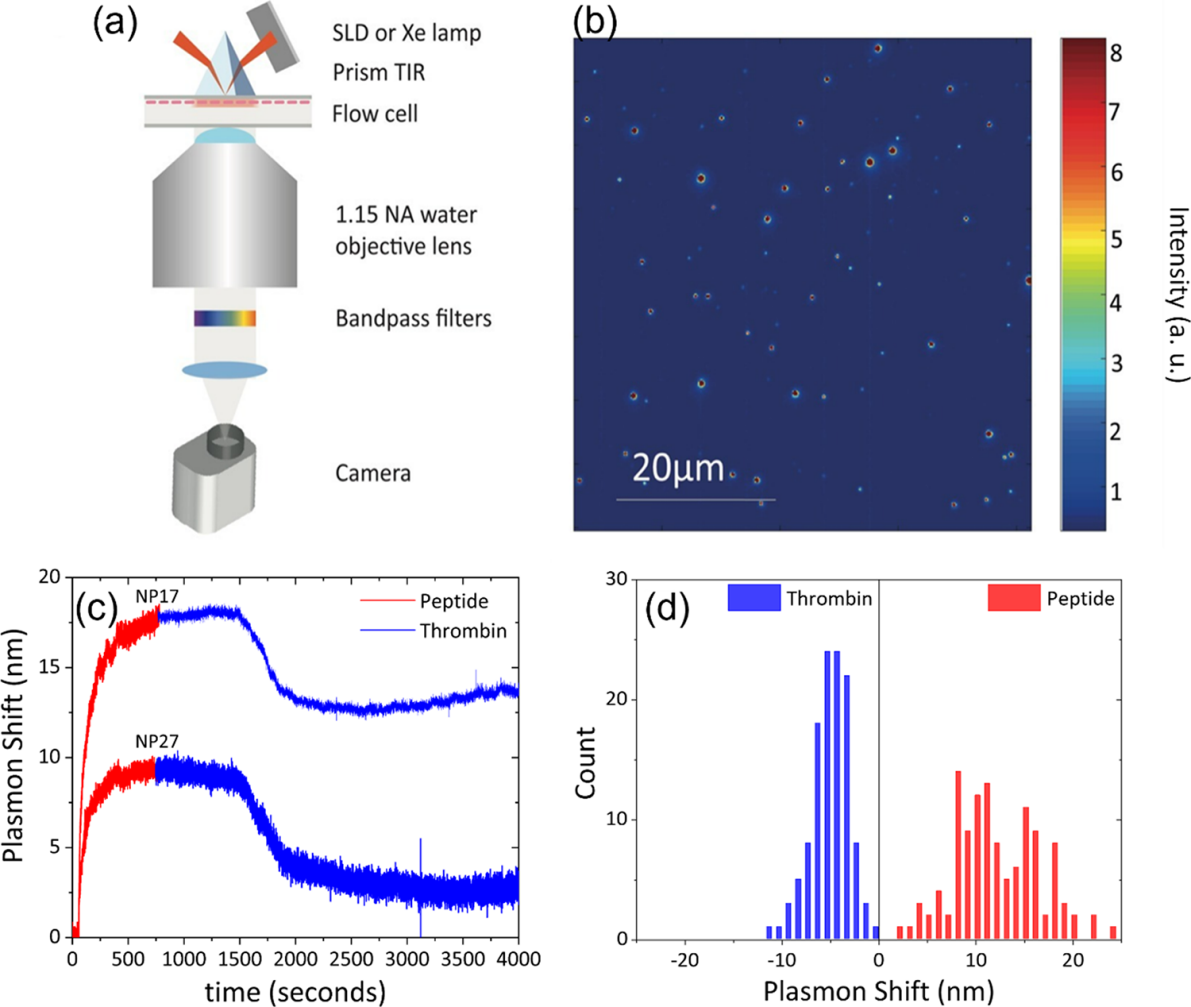
(a) Schematic representation of the dark-field microscope setup. (b) Dark-field scattering microscopy image of the AuNRs on the sample. Each spot represents light scattered by a single AuNR. (c) Representative single-particle time traces from two distinct nanoparticles (NP17 and NP27) showing the plasmon shifts during the peptide immobilization at a peptide concentration of 10 μM and the enzymatic cleavage at a thrombin concentration of 50 nM. (d) Distribution of single-particle plasmon shifts measured across one field-of-view of the microscope during peptide immobilization (10 μM peptide concentration, in red) and cleavage (50 nM thrombin concentration, in blue).

A schematic representation of the dark-field scattering setup used (based on earlier reports^12^) is depicted in Figure 2a. In short, sample illumination is achieved using total internal reflection and the scattered light from the AuNRs is collected by the objective. An exemplary image is shown in Figure 2b, where individual particles are revealed as diffraction-limited point-spread functions. As shown in previous correlative microscopy, each diffraction-limited spot corresponds to a single AuNR.^31^ Further confirmation of the presence of single AuNRs is provided by the shape of their scattering spectrum.^32^ To determine the spectrum of each AuNR, we resorted to hyperspectral microscopy (HSM) using white-light illumination. The resulting spectrum is fitted with a Lorentzian profile to get the plasmon peak position and its linewidth. Only particles with Lorentzian linewidth <200 meV were included in the analysis, as broader linewidths often indicate particle clustering. We find that ∼20% of the spots represent dimers or higher-order clusters, as indicated by a broad linewidth or the appearance of a double peak. We discard such spots from further analysis.

To probe plasmon shifts in real time, we use a superluminescent diode, centered at 793 nm wavelength, as an illumination source. In this configuration, plasmon shifts are conveyed as changes in scattered intensity because the spectral overlap between the plasmon resonance and the light source changes. The advantage of this approach is that plasmon shifts can be probed on millisecond timescales because the need for broadband spectroscopy is circumvented. Using the plasmon wavelength and linewidth from the single-particle scattering spectra of the same particles, we convert intensity to a plasmon shift (see Figure 2c and SI). This procedure is automatically performed for several hundred nanoparticles in the field-of-view, allowing for statistical analysis of the plasmon shifts as shown in Figure2d.

The peptide functionalization of the particle (red line in Figure 2c) induces a large redshift of 13 ± 4 nm, indicating that the particles were effectively functionalized. The spread in measured plasmon shift is caused by particle-to-particle differences in the peptide density and refractive-index sensitivity.^33^ Then, after immobilizing the peptide on the AuNRs, we performed the proteolytic assay by injecting thrombin into the flow cell. Two examples of typical time traces are shown as the blue line in Figure 2c, recorded on one of the particles in the field of view for 50 nM of thrombin. The plasmon resonance remains stable until the enzyme enters the reaction chamber, after which a rapid blueshift is observed. As expected, this blueshift is less pronounced than the redshift due to peptide functionalization, indicating that only part of the peptide is removed by the cleavage. The peptide cleavage resulted in an average blueshift of 5 ± 2 nm, indicating that approximately 40% of the plasmon redshift is recovered due to enzymatic cleavage. This is reasonable since the peptide was designed such that approximately half its length is cleaved off by thrombin. In all assays, we observed a strong correlation between the plasmon shifts of both the peptide immobilization and the enzymatic reaction (see Figure S1), suggesting that either particle with high peptide density are on average more responsive to peptide cleavage, or that there are differences in sensitivity among the particles.

**Figure 3:**
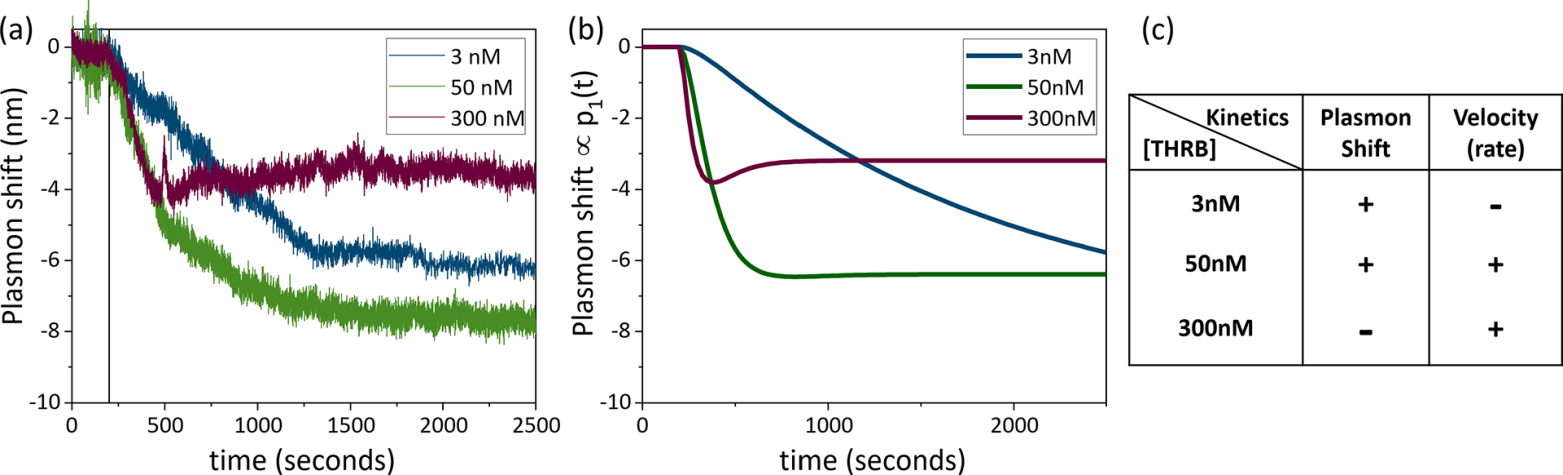
(a) Representative timetraces collected during cleavage with different concentrations of thrombin. The starting point (*t* = 0 s) of the time traces was aligned to enable comparison. (b) Kinetic curves simulated from eq. 1 using *k*_a_ = 2 × 10^5^ M^–1^ s^–1^; *k*_b_ = 0.01 s^–1^; *k*_c_ = 2.5 × 10^4^ M^–1^ s^–1^; *k*_d_ = 0.005 s^–1^; *p*_T_ = 100 and an average shift of 0.08 nm per peptide. (c) Table demonstrating the nontrivial behavior from the sensor at three different concentrations, which are separated by two orders of magnitude.

We then compared the kinetics of cleavage across different active enzyme concentrations. Characteristic time traces from three different thrombin concentrations (3, 50, and 300 nM) are presented in Figure 3a. To facilitate comparison among the kinetic profiles, we chose particles with similar LSPR (∼825 nm) while the time axis was adjusted to ensure that the enzymatic reaction starts simultaneously for all the particles (at *t* ≈ 200 s, see vertical line).

At first glance, we perceive a nontrivial behavior as the concentration of active enzymes increases (Figure 3a). At low concentrations of thrombin (3 nM, blue), we observe slow kinetics as the plasmon shift continuously evolves. For intermediate concentrations (50 nM, green), the cleavage rate increases, as well as the plasmon shift, which reaches a limit value before the end-point. In contrast, at large concentrations (300 nM, purple), even faster kinetics are observed; however, the average plasmon shift for all particles in the field-of-view is significantly smaller when compared to 3 and 50 nM thrombin. We attribute this to a competing mechanism where thrombin adsorbs nonspecifically to the particle surface. This results in a redshift of the plasmon resonance that competes with the cleavage-induced blueshift in a concentration-dependent manner. At higher concentrations (300 nM), it is possible to observe a local maximum plasmon shift (toward the blue end of the spectrum) followed by a slight shift back toward the red end. This is thought to be due to the nonspecific adsorption of thrombin, which is closely linked to the peptide cleavage step and occurs over a longer period of time. Both experimental data and the kinetic model support this explanation, with a maximum blue shift being observed after several hundred seconds and a subsequent slight red shift due to nonspecific interactions with thrombin.

To better understand the effect of these two competing mechanisms, we construct a simplified kinetic model (detailed in SI) that assumes a three-stage process: (I) first, the enzyme at concentration c_0_ adsorbs onto the peptide layer with a rate *k*_a_c_0_ without producing a measurable redshift. We base this assumption in our control experiment in the presence of a THRB inhibitor (Figure S3d) from which it is still possible to observe a slight blueshift due to incomplete inhibition of THRB, indicating that THRB does not adsorb to the intact peptide layer or it occurs in a much slower timescale (II) subsequently, the enzyme at the surface layer binds to the peptide in the target region and cleaves it at a rate *k*_b_; and (III) peptide cleavage exposes regions where later an enzyme may nonspecifically interact with rate constants *k*_c_*c*_0_ for association and *k*_d_ for dissociation, respectively. It was further assumed that the plasmon shift associated with stage I is negligible because the enzyme binds on the thick peptide layer, and that the magnitudes of blueshift and redshift from stages II and III, respectively, are similar. This assumption implies that only peptides that are exposed to solvent (i.e., without nonspecifically bound thrombin) result in a blueshift. As detailed in the SI, the time-dependent plasmon shift Δ*SP*(*t*) is then given by
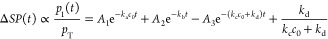
1where *p*_T_ is the total number of peptides per particle and *p*_1_ is the number of cleaved peptides that are solvent-exposed. The solution concentration of active thrombin *c*_0_ is assumed to be time-independent. The pre-exponential factors *A_i_* depend only on the rate constants previously described and are omitted here for the sake of brevity (see full details in SI). The last term in eq 1 makes it explicit that at long times, the overall plasmon shift is determined by the adsorption/desorption equilibrium on the peptide-cleaved regions.

The kinetic curves shown in Figure 3b were extracted from eq 1 where the rate constants were adjusted to match the experimental data. Despite the model’s simplicity and ensemble chemical kinetics, it accounts very well for the main features observed in the experimental time traces. The model predicts *k*_b_∼0.01 s^–1^ which is significantly slower than solution-phase rates, presumably due to steric hindrance from peptide crowding on the particle surface.

At low enzyme concentrations, the reaction rate is limited by adsorption in stage I because *k*_a_*c*_0_ ≪ *k*_b_. At *c*_0_ > 10 nM, the nonspecific adsorption in stage III starts competing with peptide cleavage, thereby decreasing the end-point plasmon shift. The model nicely accounts for a local maximum observed at *t*∼500 *s* in the experimental data for 300 nM, which occurs because the peptide cleavage rate is faster than the rate of nonspecific interactions. The best correspondence between model and experimental results was found for *k*_c_ ≪ *k*_a_, which indicates that nonspecific adsorption in stage III is less efficient than specific adsorption in stage I. This is expected because cleaved regions form an adsorption site only if their dimensions are large enough to host an enzyme (Figure S4).

**Figure 4:**
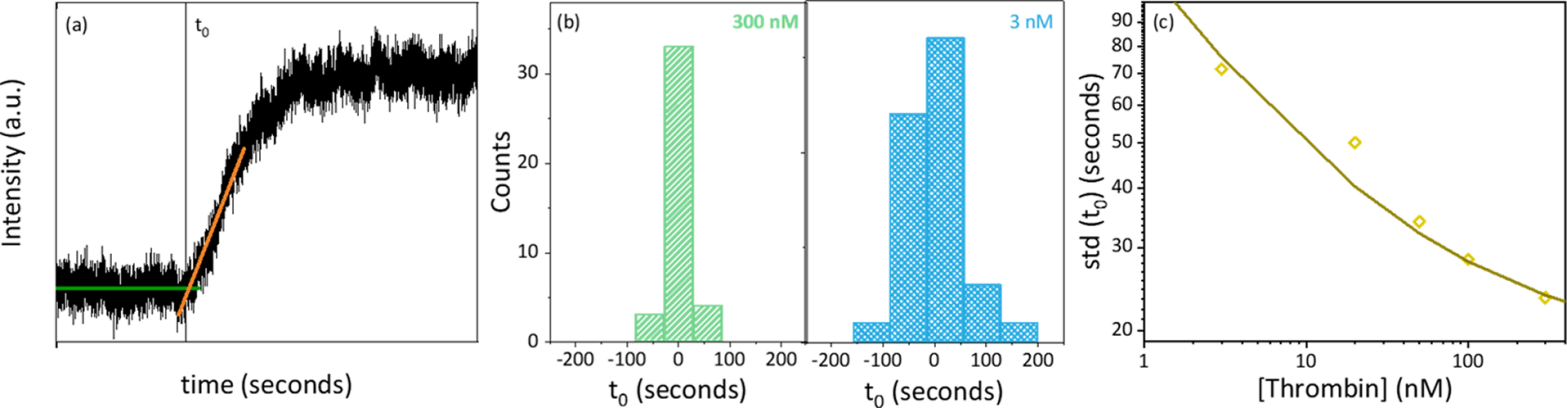
Kinetic parameters extracted from the time traces. (a) Illustration of the determination of the start time *t*_0_, determined from the intercept between the two indicated linear fits. (b) Histogram of *t*_0_ for two thrombin concentrations; (c) concentration dependence of *t*_0_. The solid line indicates a fit to the datapoints further explained in the main text (fit parameter *k*_a_ = 1 × 10^5^ M^–1^ s^–1^).

The model renders a good match with the experimental data when assuming an enzyme binding rate of *k*_a_ = 2 × 10^5^ M^–1^ s^–1^, which we independently verify by analyzing the variability in the time *t*_0_ at which the cleavage starts: the diffusion of thrombin toward the NPs’ surface is dictated by mass transport, and thus, it is stochastic. This results in particle-to-particle differences in *t*_0_, which we extracted from the intercept between two linear fits to the data, see Figure 4a. Because the absolute value of *t*_0_ depends on the exact time at which the thrombin solution enters the flowcell, we instead consider the standard deviation of the distribution of *t*_0_. As shown in Figure 4b,c, the standard deviation of *t*_0_ is reduced for higher thrombin concentrations, indicating that the time at which the first enzyme binds becomes less heterogeneous at increasing concentrations. This can be understood by considering that the rate of mass transport increases at higher concentrations, thereby decreasing the absolute waiting time until the first binding event and thus also decreasing the standard deviation of the waiting times.

The waiting time for the first enzyme to bind to a particle is dictated by the association rate *k*_a_ and the thrombin concentration *c*_0_. The average waiting time is given by τ_w_ = (*k*_a_*c*_0_)^−1^ with a standard deviation of  due to the stochastic (Poissonian) nature of the mass transport. The standard deviation *σ* was fitted to the measurements as shown in Figure 4c, where we have allowed for an additional uncertainty of 20 s in determining the starting time *t*_0_ due to the limited signal-to-noise ratio. We found a very good agreement between the assumption of Poisson statistics and our experimental data for an association rate of *k*_a_ = 1 × 10^5^ M^–1^ s^–1^. This value is in good agreement with the value found from the kinetic model fits in Figure 3.

**Figure 5:**
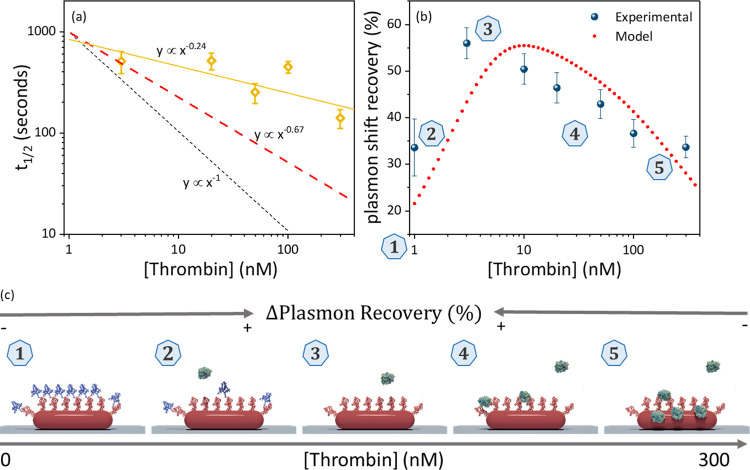
Concentration dependence results in (a) Reaction half-time of *t*_1/2_. The error bars indicate the standard deviation of the distribution in Figure S5, whereas the dashed line indicates the expected linear dependence on thrombin concentration in case mass transport dictates the cleavage rate; (b) normalized plasmon shift vs concentration of active enzyme results – experimental and modeled results; (c) illustration of the events occurring and its correlation to sensor’ performance (1 to 5). Note: an illustration of the phenomena underlying the results from our model (dashed line; red) is presented in Figure S4.

We now turn to the analysis of the cleavage rate. We observe kinetics that cannot be described by a simple single-exponential (Langmuir) model, so to extract kinetic constants, we use the reaction half-time *t*_1/2_. This represents the time at which the plasmon shift has evolved to half of its final value (see Figure S5a) and is model-independent. We find that at each thrombin concentration, the single-particle half-times are distributed normally (see Figure S5b), so in Figure 5a, we only plot their average value as a function of concentration. We find a sublinear dependence of half-time on thrombin concentration that scales approximately as *t*_1/2_ ∝ *c*_0_^–*n*^ with *n* = 0.24. The kinetic model also predicts a sublinear dependence due to the competition between peptide cleavage and nonspecific enzyme interactions, albeit with an exponent *n* = 0.67 that is closer to linearity. A sublinear sensor response was also observed in previous literature reports where end-point plasmon shifts were measured,^34^ which was attributed to crowding, steric hinderance, or enzyme depletion by nonspecific interactions that are not captured in the rate models.

After evaluation of the real-time kinetic data, we now shift our attention to the end-point plasmon shift for different concentrations of active enzyme. Because of the particle-to-particle differences in peptide loading and/or particle sensitivity, as discussed above, we normalized the plasmon blueshift to the redshift due to peptide conjugation (see Figure S6). Indeed, the concentration dependence of the maximum plasmon shift recovery correlates strongly with the thrombin concentration, see Figure 5b. As expected, the scaling is not linear due to the competition between peptide cleavage and nonspecific adsorption, as discussed above. In addition, at low concentrations of active enzyme (1 nM), the maximum contrast is not reached because a considerable portion of the peptide remains uncleaved during the measurement time. These observations are in good agreement with ensemble-averaged results using gold spheres obtained by Svärd et al.^34^ Moreover, the trend shown by our kinetic model – discussed above – matches the experimental results (Figure 5b). This indicates that the mechanisms underlying sensor response are properly explained by a competition between peptide cleavage and nonspecific enzyme binding. We speculate that these nonspecific interactions may be a consequence of the affinity of the exosite II (one of thrombin’s active sites) toward negatively charged surfaces,^35^ which become exposed upon peptide cleavage.

The presented proteolytic sensor provides a versatile plasmonic platform to study and measure proteolytic activity in real time. Thrombin activity monitoring has biological and clinical relevance in the study of coagulopathies and other diseases. To evaluate this, we benchmark the performance and the features of our sensor against thrombin generation assays (TGAs). The working range of our sensor at low nanomolar concentrations matches the relevant thrombin concentrations and its equivalent activity in pathophysiological conditions.^36^ Ultimately, other key parameters such as the lag-time and endogenous thrombin potential^37,38^ also have the potential to be determined using our plasmonic proteolytic biosensor.^39^ The presented plasmonic platform paves the way to the exploitation of the promising features of plasmonic-based proteolytic sensors in biochemical research as drug development, such as protease inhibitors (as inferred from Figure S3d), well as in diagnostics and prognostics.

## Conclusions and Outlook

A single-particle plasmonic biosensor to determine proteolytic activity in real time was developed using thrombin as a model protease. Cleavage of a thrombin-specific peptide immobilized on AuNRs enabled the label-free and real-time measurement of cleavage kinetics at the level of single particles. We found that the total amount of cleaved peptide only depends weakly on thrombin concentration, indicating a substrate-limited reaction in which all peptide eventually gets cleaved irrespective of the thrombin concentration. The sensor exhibits a dynamic range of >2 orders of magnitude with a detection limit of 3 nM while providing a stable readout over long timescales. Furthermore, by monitoring the process, we were able to extract kinetic parameters such as the binding time of the first active enzyme (*t*_0_) and the reaction half-time (*t*_1/2_). The kinetics of the sensor are in good agreement with a kinetic model that reproduces the temporal evolution of the plasmon shift as well as the end-point shift after the reaction has been completed.

Regarding thrombin, subsequent work should include the effects of external factors such as temperature, pH, and biological media on the kinetic parameters and the sensor’s potential to be used in complex samples and as TGA. Lastly, the sensor concept is easily expanded to multiplexed detection by using particles with a different LSPR. The ability to measure cleavage kinetics on each particle opens the window to the spatial mapping of protease activity in the vicinity of cells and tissue, which may lead to a deeper comprehension of metabolic processes involving proteases.

## Materials and Methods

### Materials

AuNRs with a plasmon resonance at 780 nm and stabilized in a solution containing cetyltrimethylammonium bromide (CTAB) were acquired from Nanoseedz (product #NR 10-780). The average size of the AuNRs was 39 nm × 10 nm. Figure 1 shows an electron microscope image of a dried drop of the AuNRs on a carbon-coated grid.

The peptide was acquired from Caslo ApS (Denmark), and it has the following sequence: (Biotin)-RRRGRGRGRGRGRGGDGLVPR|GSGDGGRDDRDDRDDDDDDCC-NH2. In detail, the sequence comprises biotin at the N-terminal (not used in this work), two spacer regions, a target sequence (denoted with the “|” symbol), and two cysteines to ensure strong particle-peptide conjugation via sulfur-gold adsorption.

### AuNR Immobilization

The stock solution of AuNRs (optical density 1) was diluted 50× in a 1 mM CTAB solution and subsequently centrifuged at 1500 rpm for 3 min to precipitate potential clusters. The supernatant was collected for further use.

To prepare the thiol (−SH) functionalized coverslips, we started by cleansing the surface by sonicating them in methanol for 15 min and dried using a N_2_ flow. Then, the surface was activated using an O_2_ plasma cleaner for 1 min or UV/Ozone cleaning for 90 min. Grafting of the thiol groups was achieved by immersion of the coverslips in a 5% v/v solution of mercaptopropyltrimethoxysilane (MPTMS) in absolute ethanol for 3 min and consequent rinsing with ethanol and methanol removed excess MPTMS. Lastly, sonication in methanol for 20 min and drying in a N_2_ flow were repeated.

Immediately after, to immobilize the AuNRs onto the coverslip, ∼100 μL of AuNR solution (prepared above) was drop casted and spin-coated at 2000 rpm for 90 s. The remaining CTAB was removed by rinsing with phosphate-buffered saline → Milli-Q water → Methanol, and dried using a N_2_ flow. Lastly, the sample was assembled into a flow cell (Warner instruments) and mounted under the microscope. This procedure results in well-isolated particles with ∼100 to 200 individual spots in the field-of-view of our microscope. Samples were used within 2 weeks after preparation.

### Optical Microscopy

A schematic representation of the dark-field scattering setup (based on earlier reports^30^) equipped with an autofocus system is depicted in Figure2a. In short, the setup uses total internal reflection illumination through a prism. The light scattered by the particles is collected by the water immersion objective lens (Nikon 1.15 NA CFI Apochromat LWD Lambda S 40XC) and directed to the camera (Andor Ixon 885+ EMCCD). An exemplary image is shown in Figure2b where individual particles are revealed as diffraction-limited point-spread functions.

White-light scattering spectra of the individual particles were collected using HSM using white-light illumination (Energetiq EQ-99X) through the prism.^30^ A range of bandpass filters with a 10 nm passband are introduced in the detection path, and the scattered intensity at each wavelength band is determined by a 2D Gaussian fit to the point-spread function (PSF). The resulting spectrum is fitted with a Lorentzian profile to get the plasmon peak position and its linewidth. Only particles with Lorentzian linewidth <200 meV were included in the analysis, as broader linewidths often indicate particle clustering.^44^ We find that ∼20% of the spots represent dimers or higher-order clusters, as indicated by a broad linewidth or the appearance of a double peak. We discard such spots from further analysis.

To probe plasmon shifts in real time, we use a superluminescent diode (Superlum SLD-38-HP, center wavelength 793 nm, emission bandwidth 15 nm) as an illumination source. In this configuration, plasmon shifts are conveyed to changes in scattered intensity because the spectral overlap between the plasmon resonance and the light source changes. These changes in scattered intensity are extracted by a 2D Gaussian fit of the PSF, yielding an integrated scattered intensity that is normalized to the initial intensity. Using the plasmon wavelength and linewidth from the single-particle scattering spectra of the same particles, we convert intensity to a plasmon shift (see Figure 2c and SI). This procedure is automatically performed for several hundred nanoparticles in the field-of-view, allowing for statistical analysis of the plasmon shifts as shown in Figure2d.

### Peptide Functionalization

Peptide aliquots with 100 μM concentration were prepared by resuspending the dried peptide in 25 mM citrate buffer with pH 6.5 and 50 mM NaCl. The aliquots were kept at −20 °C until use. Peptides were conjugated to the particles by first diluting the aliquots to 10 μM in a buffer containing 50 mM citric acid and 1.5 M NaCl at pH 3. A 100 μL/min flow rate was used to inject the peptide into the flow cell. The flow was continued for 10 min until the plasmon shift saturated. After this, the remaining unbound peptide was removed from the flowcell by flushing with 1.5 mL of incubation buffer.

### Thrombin Activity Assays

Active thrombin was acquired from Abcam (ab62452) and re-dispersed into 1 μM aliquots using an incubation buffer composed of 50 mM Tris–HCl, 10 mM CaCl_2_, and 150 mM NaCl at pH 8.4. Thus, aliquots with different concentrations of active thrombin (1, 3, 20, 50, 100, and 300 nM) were prepared and frozen until usage. During our experiments, thrombin’s solution was injected using a 5 μL/min flow rate and maintained throughout the measurement.

## Supporting Information Available

Correlation between the plasmon shifts induced by the peptide immobilization and the consequent enzymatic cleavage (20 nM thrombin). Dash line represents a reference line (*y* = −0.5*x*) (Figure S1); illustration of the quantities used in this derivation (Figure S2); comparison between peptide sequences and the effect of inhibition. (a) Modifications in the peptide sequence; (b) calculation of the physicochemical properties of both sequences (pepcal from Innovagen); (c) graphical representation of both peptides’ structure. (d) The effect of inhibitor in sensor’ response (Figure S3); illustration of the molecular phenomena underlying the performance of the sensor and the parameters included in the model (Figure S4). (a) and (b) Kinetic curves simulated from eq 1 of the main text using *k*_*a*_ = 2 × 105 M^–1^ s^–1^, *k*_*b*_ = 0.01 s^–1^, *k*_*c*_ = 2.5 × 104 M^–1^ s^–1^, *k*_*d*_ = 0.005 s^–1^, δ · *p*_*T*_ = 0.8 and 8, respectively, and assuming an average shift of δ = 0.08 nm per peptide. (C) Kinetic curves simulated varying δ · *p*_*T*_ values for the same concentration of active enzyme (50 nM); reaction half-time determination (a) Illustration of the determination of the half-time *t*_1/2_; (b) Histogram of *t*_1/2_ for two thrombin concentrations (Figure S5); quantification of active enzyme. Nontrivial response from plasmon shift vs concentration of active enzyme – experimental and modeled results (Figure S6); fitting results overview. Comparison of the estimated plasmon shifts and respective errors from different THRB concentrations. The average reaction time for each experiment was the following: 1 nM ∼ 80 min; 3, 10, and 20 nM ∼ 50 min; 50 nM ∼ 15 min; 100 nM ∼ 20 min; 300 nM ∼ 5 min (Table S1) (PDF)

## Abbreviations

LSPR: localized surface plasmon resonance
AuNRs: gold nanorods
DFM: dark-field microscopy
TIR: total internal reflection
THRB: thrombin
