# Supplementary material for: Single-Particle Plasmon Sensor to Monitor Proteolytic Activity in Real Time
Source: ACS Appl Opt Mater. 2023 Oct 4;1(10):1661–9. doi: 10.1021/acsaom.3c00226 (PMC10616847; doi:10.1021/acsaom.3c00226)
Supplement: Supplementary file 1 — ot3c00226_si_001.pdf [file ot3c00226_si_001.pdf]

Supplementary Information for:

A single-particle plasmon sensor to monitor proteolytic activity in real-time

Rui Oliveira-Silva[a,b,c*], Yuyang Wang[a], Sjoerd W. Nooteboom[a], Duarte M. F. Prazeres[b,c],

Pedro M. R. Paulo[d], Peter Zijlstra[a **]

[a]MBx Molecular Biosensing, Department of Applied Physics and Institute for Complex Molecular Systems, Eindhoven University of Technology, P.O. Box 513, 5600 MB, Eindhoven, The Netherlands; [b]iBB – Institute for Biotechnology and Bioengineering, Instituto Superior Técnico - Universidade de Lisboa, 1049-001 Lisboa, Portugal [c]Associate Laboratory i4HB—Institute for Health and Bioeconomy, Instituto Superior Técnico - Universidade de Lisboa, 1049-001 Lisboa, Portugal and [d]CQE - Centro de Química Estrutural, Institute of Molecular Sciences, Instituto Superior Técnico - Universidade de Lisboa, Avenida Rovisco Pais 1, 1049-001 Lisboa, Portugal

Corresponding Authors: ruipsilva@tecnico.ulisboa.pt (R. Oliveira-Silva) * and p.zijlstra@tue.nl (P. Zijlstra) **

Contents

1. ***Single-particle correlation between peptide loading and response to proteolytic activity ……………….…2***
2. ***Conversion of intensity signal to plasmon shift: Intensity-based plasmon sensing…………………….…2***
3. ***Thrombin inhibition and adsorption onto the (uncleaved) peptide layer ………………………..…...4***
4. ***Kinetic modelling: A pseudo-first order kinetic model for protease adsorption and cleavage…………..4***
5. ***Reaction half-time determination…………………………………………………………………......7***
6. ***Lorentzian derivate fitting results vs concentration of active enzyme (table)……………………….........7***
7. ***Non-trivial response of the plasmonic sensor……………………………………………………………...8***

Single-particle correlation between peptide loading and response to proteolytic activity

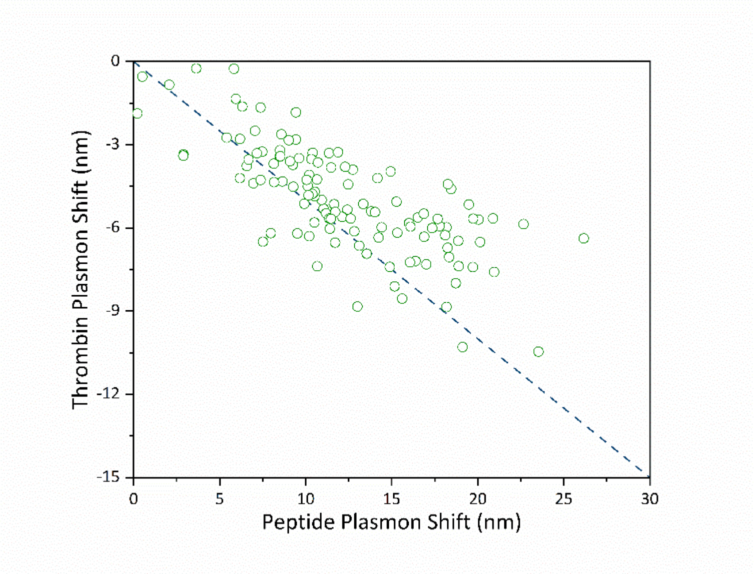

Figure S1. Correlation between the plasmons shifts induced by the peptide immobilization and the consequent wnzymatic cleavage (20nM thrombin). Dash line represents a reference line (y = -0.5x).

Intensity-based plasmon sensing

We will derive equations to convert changes in the intensity scattered by a single nanoparticle into a plasmon shift. We assume that the plasmon resonance can be approximated by a Lorentzian function given by

$$I(E) = \frac{\Gamma}{2\pi} \frac{1}{\left(E - E_{sp}\right)^2 + \left(\frac{1}{2}\Gamma\right)^2} \tag{1}$$

Where $\boldsymbol{E}$ is the incident photon energy, $\boldsymbol{E_{sp}}$ and $\boldsymbol{\Gamma}$ are the plasmon energy and linewidth (measured from the scattered spectrum). Note that all units are in eV. In a biosensing experiment this spectrum becomes time dependent due to a time-dependent shift of the plasmon resonance. This can be taken into account by considering $\boldsymbol{E_{sp}}$ to be time-dependent due to a shift $\boldsymbol{\Delta E_{sp}(t)}$. The plasmon resonance is then given by

$$I(E,t) = \frac{\Gamma}{2\pi} \frac{1 + A\Delta E_{sp}(t)}{\left(E - [E_{sp} + \Delta E_{sp}(t)]\right)^2 + \left(\frac{1}{2}\Gamma\right)^2}. \tag{2}$$

Herein the factor $\boldsymbol{A}$ has a negative value and describes the increase in scattering cross section in response to a plasmon redshift (i.e. a decrease in cross section with an increase in plasmon energy). The contrast in an intensity-based experiment is probed using a light source with a center energy $\boldsymbol{E_p}$. We assume that the linewidth of the source is much narrower than $\boldsymbol{\Gamma}$. The contrast is then given by:

$$contrast\ (t) = \frac{I(E_p, t)}{I(E_p, 0)} = \frac{[1 + A\Delta E_{sp}(t)]\left[\left(E_p - E_{sp}\right)^2 + \left(\frac{1}{2}\Gamma\right)^2\right]}{\left(E_p - [E_{sp} + \Delta E_{sp}(t)]\right)^2 + \left(\frac{1}{2}\Gamma\right)^2}. \tag{3}$$

Provided the value of A is known (either estimated from the asymmetric shape of a typical s-curve, or estimated from a numerical model of the scattering spectrum) this approach can be used to solve analytically for $\Delta E_{sp}(t)$. For $A = 0$ we can extract $\Delta E_{sp}(t)$ directly:

$$\Delta E_{sp}(t) = E_p - E_{sp} \pm \sqrt{\frac{\left(E_p - E_{sp}\right)^2 + \left(\frac{1}{2}\Gamma\right)^2}{contrast(t)} - \left(\frac{1}{2}\Gamma\right)^2}, \tag{4}$$

Where the plus sign holds when $E_p < E_{sp}$ and the minus sign when $E_p > E_{sp}$.

For $A \neq 0$ we first need to rewrite the equation into:

$$p_2 \Delta E_{sp}(t)^2 + p_1 \Delta E_{sp}(t) + p_0 = 0, \tag{5}$$

Where,

$$p_2 = contrast(t),$$

$$p_1 = 2 * contrast(t)\left[E_{sp} - E_p\right] - A\left[\left(E_p - E_{sp}\right)^2 + \left(\frac{1}{2}\Gamma\right)^2\right], \tag{6}$$

$$p_2 = \left[contrast(t) - 1\right]\left[\left(E_p - E_{sp}\right)^2 + \left(\frac{1}{2}\Gamma\right)^2\right]. \tag{7}$$

From here $\Delta E_{sp}(t)$ is easily calculated:

$$\Delta E_{sp}(t) = \frac{-p_1 \pm \sqrt{(p_1^2 - 4p_2 p_0)}}{2p_2}, \tag{8}$$

Where the plus sign holds when $E_p < E_{sp}$ and the minus sign when $E_p > E_{sp}$.

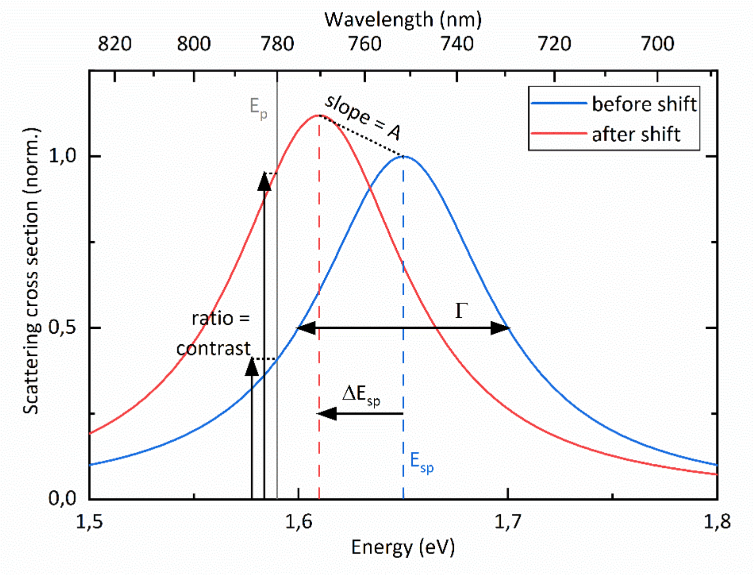

Figure S2. Illustration of the quantities used in this derivation.

Thrombin inhibition and adsorption onto the (uncleaved) peptide layer

Firstly, it is important to note that due to difficulties from the manufacturing company (CASLO) in reproducing the original peptide (due to Aspartimide formation) the original sequence was slightly modified. The modification involves exchanging part of the aspartic acids (D) for glutamic acid (E) (Figure S3a) aiming to cause minimum interference in the peptide as possible. Consequently, the physicochemical properties of both sequences, such as hydrophobicity, charge and isoelectric point (pI), were estimated using the bioinformatic tool Pepcalc from Innovage. From Figure S3b, as expected, it is possible to observe that both sequences have similar physicochemical properties being the overall net charge of the peptide across the pH values and the hydropathy equal to both peptides and only slight change in the isoelectric point (from 6.32 to 6.45) was expected. Further, we assessed eventual conformation changes between both peptide sequences using the AlphaFold software through COLAB [1] project using ChimeraX software to predict the structures of the peptide sequences. For visual comparison and alignment, the PDB files were imported to Blender v3.3 using the Molecular Nodes add-on from which it can be observed that both peptides present a similar “random coiled” structure (Figure S3c)

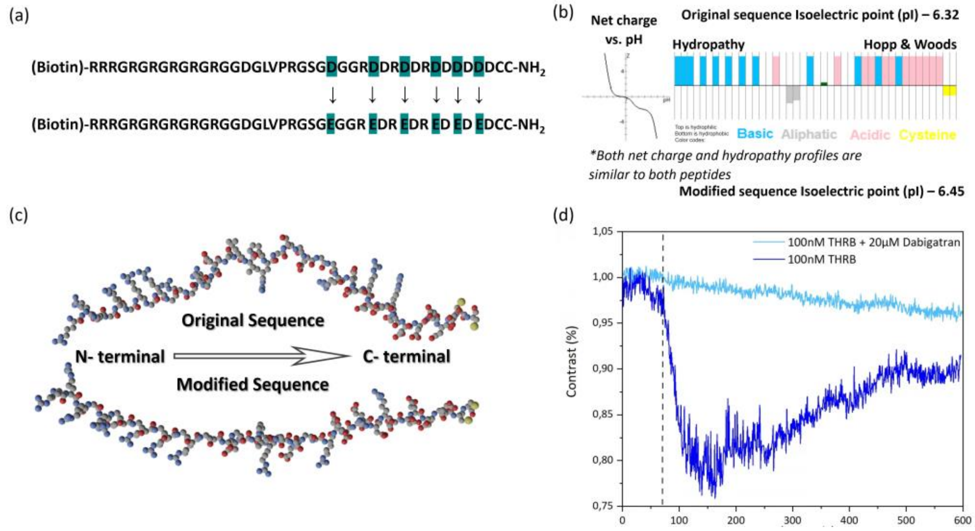

Figure S3. Comparison between peptide sequences and the effect of inhibition. (a) Modifications in the peptide sequence; (b) Calculation of the physicochemical properties of both sequences (pepcal from Innovagen); (c) Graphical representation of both peptides’ structure. (d) The effect of inhibitor in sensor’ response.

After comparison of both peptide sequences using bioinformatic tools, we performed an experiment to evaluate how the presence of an well-known inhibitor (Dabigatran; 20µM) would influence the response of our sensor using AuNRs with 25nm width with LSPR at 650nm while probing them at 640nm±15nm. Hence, Figure S3d are provided the timetraces for an experiment using 100nM THRB and 100nM THRB and inhibitor. Here, to provide an accurate and broad impression of both experiments, the timetraces of several particles were averaged before plotting. As expected, in the absence of the inhibitor a quick blue-shift is observed indicating the cleavage of peptide layer by THRB. The consequent red-shift is indicative of THRB to the cleaved layer (later explained in the maintext). Conversely, in the presence of the inhibitor, when THRB enters the chamber (vertical dashed line) the sudden blue-shift is no longer observed and only occurs slowly over a long period of time, which indicates that the enzyme is not fully inhibited under these concentrations of Dabigatran.

A pseudo-first order kinetic model for protease adsorption and cleavage

The kinetic model described in the main text comprises 3 stages: (I) the enzyme adsorption onto the peptide layer; (II) the enzyme binding to the cleavage site and peptide cutting; (III) the enzyme adsorption onto cleaved regions in the peptide layer. The first and third stages are modeled as pseudo-first order reversible reactions for the adsorption/desorption equilibrium, whereas the second stage is modeled as two consecutive irreversible steps,

$$p_0 \underset{k_e}{\overset{k_a c_0}{\rightleftarrows}} p_{0a} \xrightarrow{k_b} p_{0b} \xrightarrow{k_{cat}} p_1 \underset{k_d}{\overset{k_c c_0}{\rightleftarrows}} p_{1a} \tag{9}$$

where p_0 and p_{0a} represent the intact peptide before and after enzyme adsorption, respectively, p_{0b} is the peptide with an enzyme specifically bound to its cleavage site, and p_1 and p_{1a} are the cleaved peptide without and with an enzyme (non-specifically) adsorbed, respectively. The pseudo-first order rate constants are indicated in the reaction scheme of eq. 9, where c_0 is the protease bulk concentration. When solving the kinetic equations, it was assumed that the peptide cutting rate constant k_{cat} is much larger than the binding rate constant k_b, so that p_{0b} converts almost instantaneously into p_1. The typically large values of k_{cat} for thrombin make this assumption reasonable. Whereas enzyme binding rates k_b should be fast in bulk solution because the species involved are freely diffusing, the same does not necessarily apply at the particle's surface due to molecular crowding in the peptide layer. Under these assumptions, the set of differential equations from the reaction scheme of eq. 9 gives the following solution for the time evolution of cleaved peptide,

$$p_1(t) = p_T \frac{k_a c_0 k_b}{\lambda_+ - \lambda_-} \times \left\{ \left(1 + \frac{k_d}{\lambda_+}\right) \times \frac{e^{\lambda_+ t} - e^{-k_+ t}}{\lambda_+ + k_+} - \left(1 + \frac{k_d}{\lambda_-}\right) \times \frac{e^{\lambda_- t} - e^{-k_+ t}}{\lambda_- + k_+} + \frac{k_d}{k_+} \frac{\lambda_+ - \lambda_-}{\lambda_+ \lambda_-} \times \left(1 - e^{-k_+ t}\right) \right\} \tag{10}$$

where p_T is the average number of peptides per particle. The following definitions were introduced for grouping the rate constants,

$$k_- = k_a c_0 + k_e + k_b \quad , \quad k_+ = k_c c_0 + k_d \tag{11}$$

$$\lambda_\pm = -k_-/2 \pm \sqrt{(k_-/2)^2 - k_a c_0 k_b} \tag{12}$$

The plasmon shift is roughly proportional to the number of cleaved peptides that are solvent exposed (p_1), because it is assumed that the plasmon shift associated with stage I is negligible and that the magnitudes of blue- and red-shift from stages II and III are comparable to each other. The kinetic law of eq. 1 in the main text was derived from eq. 10 by further assuming that $k_e \ll k_a c_0, k_b$ which, despite the loss of generality, still affords a good description of our experimental results. The pre-exponential factors of eq. 1 from the main text (A_i) are then given by,

$$A_1 = \frac{k_b}{k_a c_0 - k_b} \times \frac{k_d - k_a c_0}{k_+ - k_a c_0} \tag{13}$$

$$A_2 = \frac{k_a c_0}{k_b - k_a c_0} \times \frac{k_d - k_b}{k_+ - k_b} \tag{14}$$

$$A_3 = A_1 + A_2 + \frac{k_d}{k_c c_0 + k_d} \tag{15}$$

Using this simplified model, a set of kinetic curves were simulated for the values of thrombin concentration used in the experiments, as shown in fig. S1.

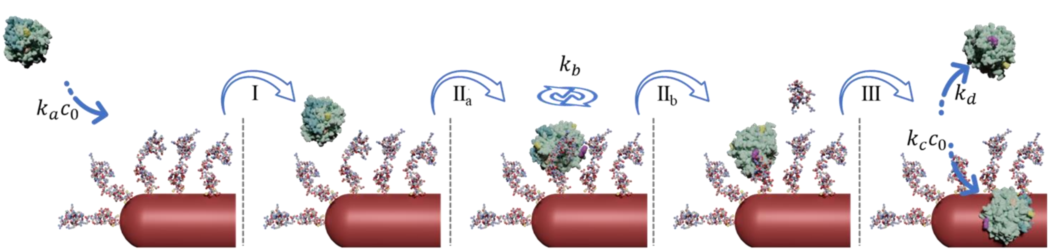

$$\Delta SP(t) \propto \frac{p_1(t)}{p_T} = A_1 e^{-k_a c_0 t} + A_2 e^{-k_b t} - A_3 e^{-(k_c c_0 + k_d)t} + \frac{k_d}{k_c c_0 + k_d}$$

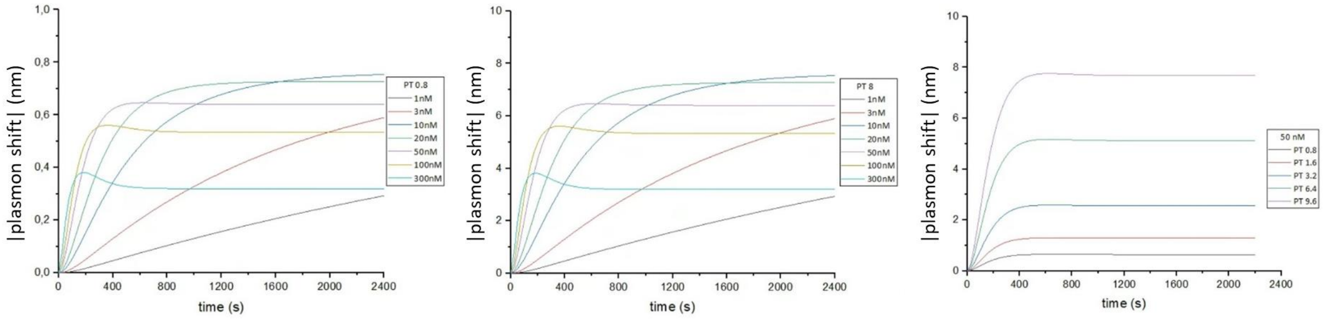

Figure S4. Illustration of the molecular phenomena underlying the performance of the sensor and the parameters included in the model. (a) and (b) Kinetic curves simulated from eq. 1 of the main text using k_a= 2×10^5 M^{-1}s^{-1}, k_b= 0.01 s^{-1}, k_c= 2.5×10^4 M^{-1}s^{-1}, k_d= 0.005 s^{-1}, $\delta \cdot p_T$= 0.8 and 8, respectively, and assuming an average shift of δ = 0.08 nm per peptide. (C) Kinetic curves simulated varying $\delta \cdot p_T$ values for the same concentration of active enzyme (50nM)

Above some concentration c_0, the simulated kinetic curves display a local maximum, which is also observed in the experimental time traces. The instant $t_{\max}$ at which this local maximum occurs can be approximately found from,

$$\left.\frac{dp_1(t)}{dt}\right|_{t_{max}} = 0 \xrightarrow{|\lambda_-| \gg |\lambda_+|\,,\ k_+} \ldots$$

$$t_{max} \approx \frac{1}{\lambda_- + k_+} \times ln\left\{\frac{k_+}{\lambda_-} \times \frac{\lambda_- + k_d}{\lambda_- + k_+} - \frac{k_+}{\lambda_+} \times \frac{\lambda_+ + k_d}{\lambda_+ + k_+} - k_d \frac{\lambda_+ - \lambda_-}{\lambda_+ \lambda_-}\right\} \tag{16}$$

which has only physical meaning for positive values of $t_{\max}$, and this sets a minimum concentration value needed to verify this condition,

$$c_0^* = \frac{k_d}{|k_a - k_b|} \tag{17}$$

In the example of Figure S3, this concentration limit is c_0^* = 28.6 nM. Instead of using the approximation of eq. 16, the values of $t_{\max}$ were numerically calculated using the method of Newton-Raphson. In order to replicate the experimental results, the model estimates of plasmon shift were calculated from $p_1(t_{\max})$, when $t_{\max} < t_{\mathrm{w}}$, when $t_{\max}$ occurs before the end point of the measurement window which was set at $t_{\mathrm{w}} = 2400$ s. Otherwise at low c_0 values, when a local maximum is not observed, the plasmon shifts were calculated at the end point of kinetic curves, i.e. from $p_1(t_{\mathrm{w}})$. This calculation afforded the model curve shown below in Figure S5, from which it was calculated the normalized plasmon shift shown in Figure 5 of the main text (red dashed curve).

Another parameter from the kinetic curves that was assessed both experimentally and theoretically was the reaction half-time $t_{1/2}$, which is defined as,

$$p_1(t_{1/2}) = p_1(t_w)/2$$

(18)

When using eq. 10 to solve eq. 18, it yields an equation that does not have an exact algebraic solution, so it was again numerically calculated using the method of Newton-Raphson. The main result is shown as a dashed red curve in Figure 5a of the main text.

Reaction half-time determination

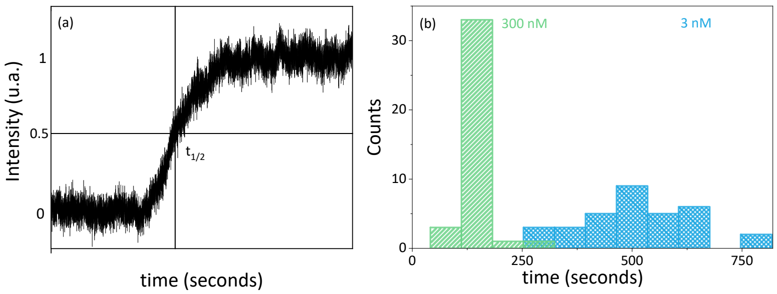

Figure S5. Reaction Half-time determination a) Illustration of the determination of the half-time $t_{1/2}$; b) Histogram of $t_{1/2}$ for two thrombin concentrations.

Lorentzian derivate fitting results vs concentration of active enzyme

Table S1. Fitting results overview. Comparison of the estimated plasmon shifts and respective errors from different THRB concentrations. The average reaction time for each experiment was the following: 1nM ~80min; 3, 10 and 20nM ~50 min; 50nM ~15 min; 100nM ~20 min; 300 nM ~5min.

[THRB]	Peptide Plasmon Shift (nm)	Fitting R^2	THRB Plasmon Shift (nm)	Fitting R^2	Plasmon shift recovery (%)	Ratio uncertainty (%)
1	13.4 ± 1	0.95	4.4 ± 0.8	0.77	32.8	6.4
3	13.4 ± 0.7	0.94	7.5 ± 0.5	0.96	56.0	4.7
10	12.3 ± 0.8	0.93	6.2 ± 0.4	0.95	50.4	4.6
20	15.3 ± 1.2	0.9	7.1 ± 0.5	0.95	46.4	4.9
50	13.0 ± 0.8	0.93	5.8 ± 0.5	0.88	44.6	4.2
100	13.3 ± 0.8	0.94	5.4 ± 0.2	0.97	40.6	2.9
300	14.7 ± 0.9	0.96	5.0 ± 0.4	0.94	34.0	3.4

Non-trivial response of the plasmonic sensor

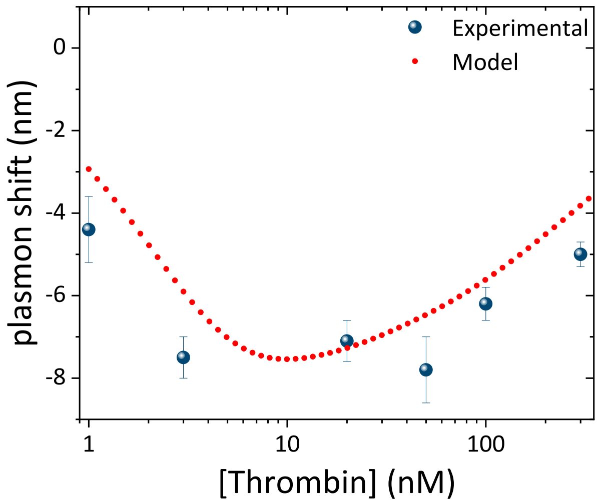

Figure S6. Quantification of active enzyme. Non-trivial response from plasmon shift vs concentration of active enzyme – experimental and modeled results.

Refences Supporting info

(1) Mirdita, M.; Schütze, K.; Moriwaki, Y.; Heo, L.; Ovchinnikov, S.; Steinegger, M. ColabFold: Making Protein Folding Accessible to All. *Nat Methods* **2022**, *19* (6), 679–682. https://doi.org/10.1038/s41592-022-01488-1.
